# Supplementary material for: High PD-L1/IDO-2 and PD-L2/IDO-1 Co-Expression Levels Are Associated with Worse Overall Survival in Resected Non-Small Cell Lung Cancer Patients
Source: Genes (Basel). 2021 Feb 15;12(2):273. doi: 10.3390/genes12020273 (PMC7918978; doi:10.3390/genes12020273)
Supplement: Supplementary file 1 [file genes-12-00273-s001.pdf]

Table S1. Univariate and multivariate analyses for recurrence-free survival (RFS) in adenocarcinomas (N.120)

			RFS Adenocarcinomas					
			Univariate Analysis			Multivariate Analysis		
**Variables**		At risck	HR	95% CI	* *p*	HR	95% CI	* *p*
**Age**								
	<60	26						
	>=60	94	0.94	0.51–1.71	0.84			
**Sex**								
	F (Ref)	40						
	M	80	1.07	0.62–1.84	0.78			
**Smoking**								
	Never (Ref)	15	1					
	Current	44	2.67	1.02–6.94	**0.04**	3.26	1.15–9.22	**0.02**
	Former	61	1.76	0.68–4.57	0.24	1.69	0.69–4.61	0.30
**p-Stage**								
	I (Ref)	73	1					
	II	26	2.14	1.15–3.98	**0.01**	2.63	1.33–5.20	**0.005**
	III	21	1.93	1.02–3.64	**0.04**	2.09	1.05–4.15	**0.03**
**PD-1**								
	Low <4fc (Ref)	104						
	High ≥ 4fc	16	1.21	0.72–2.02	0.46			
**PD-L1**								
	Low <4fc (Ref)	52						
	High ≥ 4fc	68	1.45	0.95–2.21	0.08			
**PD-L2**								
	Low <4fc (Ref)	72						
	High ≥ 4fc	48	1.80	1.08–3.00	**0.02**			
**IDO-1**								
	Low <4fc (Ref)	72						
	High ≥ 4fc	48	1.45	0.87–2.41	0.15			
**IDO-2**								
	Low <4fc (Ref)	95						
	High ≥ 4fc	25	2.24	1.27–3.94	**0.005**			
**INFγ**								
	Low <4fc (Ref)	100						
	High ≥ 4fc	20	2.09	1.14–3.81	**0.01**			
**PD-L1/IDO-2**								
	Low <4fc (Ref)	101						
	High ≥ 4fc	19	2.27	1.22–4.22	**<0.001**			
**PD-L2/IDO-1**								
	Low <4fc (Ref)	90						
	High ≥ 4fc	30	2.07	1.22–3.55	**0.04**			
**PDL1/PDL2/INFγ**								
	Low <4fc (Ref)	102						
	High ≥ 4fc	18	2.43	1.30–4.51	**0.005**			

Abbreviations: HR, Hazard Ratio; CI, Confidence Interval PD-1, programmed cell death 1; PD-L1, programmed cell death 1 ligand 1; PD-L2, programmed cell death 1 ligand 2; IDO-1, indoleamine 2,3-dioxygenase 1; IDO-2, indoleamine 2,3-dioxygenase 2; INFγ, Interferon-γ; significant *p* value ≤ 0.05 in bold.

Table S2. Univariate and multivariate analyses for overall survival (OS) in adenocarcinomas (N.120)

			OS Adenocarcinomas					
			Univariate Analysis			Multivariate Analysis		
**Variables**		At risck	HR	95% CI	* *p*	HR	95% CI	* *p*
**Age**								
	<60	26						
	>=60	94	0.94	0.51–1.71	0.84			
**Sex**								
	F (Ref)	40						
	M	80	1.84	1.02–3.30	**0.04**	1.90	1.05–3.46	**0.03**
**Smoking**								
	Never (Ref)	15	1					
	Current	44	1.90	0.77–4.67	0.15			
	Former	61	1.64	0.68–3.93	0.26			
**p-Stage**								
	I (Ref)	73	1					
	II	26	1.75	0.95–3.24	0.07			
	III	21	1.93	0.90–3.18	0.10			
**PD-1**								
	Low <4fc (Ref)	104						
	High ≥ 4fc	16	1.70	0.86–3.36	0.12			
**PD-L1**								
	Low <4fc (Ref)	52						
	High ≥ 4fc	68	1.70	1.01–2.87	**0.04**			
**PD-L2**								
	Low <4fc (Ref)	72						
	High ≥ 4fc	48	1.60	0.96–2.66	0.06			
**IDO-1**								
	Low <4fc (Ref)	72						
	High ≥ 4fc	48	1.64	0.99–2.72	**0.05**			
**IDO-2**								
	Low <4fc (Ref)	95						
	High ≥ 4fc	25	1.54	0.85–2.76	0.14			
**INFγ**								
	Low <4fc (Ref)	100						
	High ≥ 4fc	20	1.72	0.93–3.19	0.08			
**PD-L1/IDO-2**								
	Low <4fc (Ref)	101						
	High ≥ 4fc	19	1.98	1.07–3.67	**0.02**			
**PD-L2/IDO-1**								
	Low <4fc (Ref)	90						
	High ≥ 4fc	30	2.24	1.32–3.79	**0.003**			
**PDL1/PDL2/INFγ**								
	Low <4fc (Ref)	102						
	High ≥ 4fc	18	1.92	1.02–3.63	**0.04**			

Abbreviations: HR, Hazard Ratio; CI, Confidence Interval; PD-1, programmed cell death 1; PD-L1, programmed cell death 1 ligand 1; PD-L2, programmed cell death 1 ligand 2; IDO-1, indoleamine 2,3-dioxygenase 1; IDO-2, indoleamine 2,3-dioxygenase 2; INFγ, Interferon-γ; significant *p* value ≤ 0.05 in bold.
